# Supplementary material for: The impact of the #MeToo movement on language at court A text-based causal inference approach
Source: PLoS One. 2024 May 15;19(5):e0302827. doi: 10.1371/journal.pone.0302827 (PMC11095728; doi:10.1371/journal.pone.0302827)
Supplement: S1 Appendix — (PDF) [file pone.0302827.s001.pdf]

The impact of the #MeToo movement on language at court A text-based causal inference approach

Henrika Langen*

Department of Economics, University of Fribourg, Switzerland

* rikalangen@t-online.de

Additional Information on Text Processing

Legal terms for classifying judicial opinions

The legal terms for sexual offenses differ strongly across U.S. states (see https://apps.rainn.org/policy/#report-generator for state-specific terms and definitions); therefore, different state-specific sets of legal terms are used. The legal terms for offenses of interpersonal violence are more homogeneous across the states; consequently, the same set of terms is used for all courts where the legal terms are obtained from the following site: https://www.criminaldefenselawyer.com/topics/crimes-against-persons)

Legal terms for classifying judicial opinions

To evaluate the performance of this procedure, I reviewed 100 randomly selected opinions from the pool of 43,088. Among these, 91 opinions were identified as addressing crimes against persons, six dealt with parental rights, one involved a defamation lawsuit, another one a product liability claim, and one was related to firearm possession.

List of stopwords used for text vectorization (nltk.corpus stopword list and months)

a, i, only, isn't, from, monday, will, doesn't, mightn, other, ours, hasn't, when, down, the, of, your, hasn, yours, wouldn't, you'd, you're, herself, saturday, ma, won, been, you, does, wouldn, june, tuesday, shan, can, won't, on, july, theirs, until, their, doing, off, up, how, don't, not, mustn, ll, didn't, this, her, november, why, those, am, shan't, it, haven't, have, couldn, few, s, that, be, were, having, if, through, with, aren, no, re, while, just, into, doesn, both, further, in, more, wasn't, october, who, itself, thursday, themselves, m, she's, he, t, august, me, yourself, january, because, hers, needn't, ain, during, february, should, should've, shouldn't, they, then, y, we, yourselves, once, where, after, his, by, mightn't, its, now, mustn't, wasn, weren't, don, couldn't, may, to, was, same, as, shouldn, myself, are, so, these, is, you've, again, had, too, sunday, my, wednesday, some, she, needn, an, above, has, our, it's, which, out, about, very, for, what, isn, didn, between, nor, at, whom, here, hadn't, that'll, under, and, against, hadn, each, haven, but, friday, than, ve, below, most, d, march, did, over, all, do, such, there, ourselves, aren't, september, him, before, any, or, you'll, own, himself, being, april, december, them, weren, o

Identification of authoring judges of sexual offense opinions

For 9 courts, accounting for roughly 30% of the opinions in my sample, the names of the judge who wrote the opinion is provided in the CourtListener database. For the remaining sexual offense opinions, I obtain the judge's name based on a semi-automatic approach, i.e., by formulating court-specific regex-rules to identify the authoring judge, which I then check manually based on the context from which they were drawn. In cases where more than one judge is named as the author of an opinion, the name mentioned first is selected as the authoring judge, as is the case in the CourtListener database. Opinions written per curiam, i.e., in the name of the court rather than the judge(s), as well as opinions in which no authoring judge is named are excluded from the sample in the event study setting (they account for less than 1% of sexual offense opinions).

The names of the so identified judges are cleaned up, i.e., spellings of the same name and title in opinions of the same court are aligned. Of course, the entire process of identifying the authoring judge has many potential sources of error. For one thing, there could be two judges with the same name in the same court whose opinions will be attributed to one and the same individual. Second, when an opinion is authored by more than one judge, the order in which the judges are named does not necessarily say anything about the writing share of the judges, thus the judge named first and selected by me is not necessarily the primary author. It can be stated, however, that in the vast majority of opinions one single judge is named as the author and opinions with more than one authoring judge are the exception. Finally, both, the judge names identified by CourtListener and those identified by me may occasionally be incorrect. However, since there is no reason to believe that the number of erroneous judge names is time-dependent, this solely affects the estimation by introducing some additional noise, but does not lead to a systematic bias in the estimators.

SentiWordNet 3.0

The English SentiWordNet 3.0 contains more than 100,000 words, each of which is assigned sentiment scores for positivity and negativity. Since many words have different meanings/senses depending on the context in which they are used, the SentiWordNet dataset contains a separate entry for each meaning of a word. The different word meanings are ranked according to how frequently the word is used with the different meanings.

Word2Vec

Word2Vec is a method for vectorizing words in such a way that each word vector (of predefined length) captures the context in which the represented word usually appears, and thereby its semantic and syntactic properties. There are two approaches to learning the vector representation for each word in a text corpus. In the Common Bag of Words (CBOW) approach, the Word2Vec neural net takes context words (the words surrounding the unknown target word in a sentence) as input and returns probabilities for each word in the model vocabulary to appear in the given context. In the Skip-Gram approach, the neural network takes single words as inputs and predicts their context. In both approaches, the inputs are passed through a hidden layer that is constantly updated in order to optimize the returned prediction probabilities. Once the model is trained, the context vectors can be extracted from the hidden layer.

Stanford PoS Tagger

The Stanford PoS Tagger is a probabilistic conditional log-linear model that - based on lexical features of words as well as the context in which they appear - tags each word in a text as corresponding to a grammatical category such as verb, noun, proper name, etc.
