# Supplementary material for: The impact of the #MeToo movement on language at court A text-based causal inference approach
Source: PLoS One. 2024 May 15;19(5):e0302827. doi: 10.1371/journal.pone.0302827 (PMC11095728; doi:10.1371/journal.pone.0302827)
Supplement: S1 Table — Number of opinions per court. (PDF) [file pone.0302827.s002.pdf]

Descriptives

Court	# Non-sexual Offenses	# Sexual Offenses
Appellate Court of Illinois	668	192
Army Court of Criminal Appeals	10	46
California Court of Appeal	912	361
Commonwealth Court of Pennsylvania	247	114
Connecticut Appellate Court	436	164
Court of Appeals for the D.C. Circuit	60	24
Court of Appeals for the Eighth Circuit	349	194
Court of Appeals for the Eleventh Circuit	179	64
Court of Appeals for the Federal Circuit	14	3
Court of Appeals for the Fifth Circuit	259	108
Court of Appeals for the First Circuit	147	70
Court of Appeals for the Fourth Circuit	167	61
Court of Appeals for the Ninth Circuit	242	98
Court of Appeals for the Second Circuit	115	44
Court of Appeals for the Seventh Circuit	367	134
Court of Appeals for the Sixth Circuit	188	95
Court of Appeals for the Tenth Circuit	137	51
Court of Appeals for the Third Circuit	98	36
Court of Appeals of Alaska	47	31
Court of Appeals of Arizona	43	31
Court of Appeals of Arkansas	192	156
Court of Appeals of Georgia	403	375
Court of Appeals of Iowa	1244	680
Court of Appeals of Kansas	48	47
Court of Appeals of Minnesota	1	19
Court of Appeals of Mississippi	426	197
Court of Appeals of North Carolina	274	147
Court of Appeals of Tennessee	121	85
Court of Appeals of Texas	4082	2576
Court of Appeals of Virginia	92	39
Court of Appeals of Washington	204	137
Court of Criminal Appeals of Tennessee	1949	797
Court of Criminal Appeals of Texas	252	120
District Court of Appeal of Florida	1123	324
District Court, District of Columbia	366	133
District of Columbia Court of Appeals	154	34
Idaho Court of Appeals	48	42
Indiana Court of Appeals	1781	1146
Massachusetts Appeals Court	45	98
Michigan Court of Appeals	85	67
Missouri Court of Appeals	354	285
Navy-Marine Corps Court of Criminal Appeals	76	252
Nebraska Court of Appeals	99	113
New Jersey Superior Court	65	35
New Mexico Court of Appeals	60	33
New York Court of Appeals	93	40
Ohio Court of Appeals	3283	2091
Superior Court of Delaware	159	36
Superior Court of Pennsylvania	5995	3246
United States Air Force Court of Criminal Appeals	3	28
United States Court of Federal Claims	19	8

Table 1: Number of opinions per court.
