# Supplementary material for: The impact of the #MeToo movement on language at court A text-based causal inference approach
Source: PLoS One. 2024 May 15;19(5):e0302827. doi: 10.1371/journal.pone.0302827 (PMC11095728; doi:10.1371/journal.pone.0302827)
Supplement: S2 Table — Composition of sexual-violence related opinions, by crime type & year. (PDF) [file pone.0302827.s003.pdf]

Descriptives

	2015	2016	2017	2018	2019	2020
Share of Sexual Offense Opinions	0.361	0.356	0.353	0.353	0.357	0.352
		(0.503)	(0.646)	(0.865)	(0.888)	(0.275)
Composition of Sexual Offenses:						
Sexual Assault	0.834	0.815**	0.786	0.817	0.824	0.827*
		(0.932)	(0.015)	(0.644)	(0.773)	(0.098)
Sexual Assault on a Minor/Child	0.028	0.028	0.028	0.032	0.026	0.036
		(0.745)	(0.26)	(0.502)	(0.506)	(0.345)
Statutory Sexual Assault	0.134	0.151	0.148	0.141	0.134	0.155
		(0.982)	(0.616)	(0.977)	(0.42)	(0.731)
Sodomy	0.091	0.114	0.102	0.084	0.103	0.113
		(0.034)	(0.316)	(0.964)	(0.639)	(0.361)
Fondling	0.306	0.315	0.325	0.339	0.32	0.338
		(0.483)	(0.533)	(0.241)	(0.49)	(0.454)
Sexual Harassment	0.002	0.006	0.007	0.006	0.004	0.006
		(0.57)	(0.405)	(0.553)	(0.846)	(0.438)
# Opinions (Total)	6474	7685	7111	7223	7883	6713
# Sexual Offense Opinions	2333	2737	2510	2548	2812	2367

Table 1: Shares of sexual-violence related opinions that deal with different crime types (by year), as well as the p-value for the differance between the 2015 share and the share in the respective year (with court fixed effects). Note: For 2016-2020, the year y is defined as November y-1 to October y in order to have a clear cut at the onset of the #MeToo movement in November 2017; the year 2015 only consists of the months January-October 2015. Significance levels: * $p<0.1$, ** $p<0.05$, *** $p<0.01$.
