# Supplementary material for: The impact of the #MeToo movement on language at court A text-based causal inference approach
Source: PLoS One. 2024 May 15;19(5):e0302827. doi: 10.1371/journal.pone.0302827 (PMC11095728; doi:10.1371/journal.pone.0302827)
Supplement: S3 Table — Victim blaming indicators. (PDF) [file pone.0302827.s004.pdf]

DiD: Robustness Checks

Victim Blaming Indicators

	Victim as Subject			Neg. of Offender Context		
	(1)	(2)	(3)	(1)	(2)	(3)
sex cr. x placebo	-0.716			-0.012		
	(0.810)			(0.066)		
sex cr. x post		-0.939			-0.100*	
		(0.926)			(0.058)	
sex cr. x post			1.399			-0.093**
			(1.564)			(0.042)
post	X	X	X	X	X	X
court FE	X	X	X	X	X	X
# words	X	X	X	X	X	X

Table 1: Robustness tests: (1) DiD was performed using only pretreatment observations and a placebo treatment in the middle of the pretreatment period; (2) DiD with IPW based on court distribution, performed for the entire sample using the `didweight` function from the `causalweight` package in the statistical software R (?); and (3) DiD performed only for the sample of sodomy and sexual assault cases. Significance levels: * p<0.1, ** p<0.05, *** p<0.01.
