# Supplementary material for: The impact of the #MeToo movement on language at court A text-based causal inference approach
Source: PLoS One. 2024 May 15;19(5):e0302827. doi: 10.1371/journal.pone.0302827 (PMC11095728; doi:10.1371/journal.pone.0302827)
Supplement: S4 Table — Text vectorization. (PDF) [file pone.0302827.s005.pdf]

DiD: Robustness Checks

Text Vectorization

	BoW			Reduced Sample BoW			tf-idf		
	(1)	(2)	(3)	(1)	(2)	(3)	(1)	(2)	(3)
sex cr. x placebo	0.220			0.166			-0.941		
	(0.584)			(0.675)			(0.831)		
sex cr. x post		0.535			0.442			1.697**	
		(0.424)			(0.438)			(0.821)	
sex cr. x post			0.806			0.836*			4.148*
			(0.534)			(0.505)			(2.254)
post	X	X	X	X	X	X	X	X	X
court FE	X	X	X	X	X	X	X	X	X
# words	X	X	X	X	X	X	X	X	X

Table 1: Robustness tests: (1) DiD was performed using only pretreatment observations and a placebo treatment in the middle of the pretreatment period; (2) DiD with IPW based on court distribution, performed for the entire sample using the `didweight` function from the `causalweight` package in R; and (3) DiD performed only for the sample of sodomy and sexual assault cases. The distance of opinions to the H1 2015 average is expressed relative to the median distance of all opinions to the H1 2015 average, in percent. Significance levels: * $p<0.1$, ** $p<0.05$, *** $p<0.01$.
