# Supplementary material for: The impact of the #MeToo movement on language at court A text-based causal inference approach
Source: PLoS One. 2024 May 15;19(5):e0302827. doi: 10.1371/journal.pone.0302827 (PMC11095728; doi:10.1371/journal.pone.0302827)
Supplement: S5 Table — Victim blaming indicators. (PDF) [file pone.0302827.s006.pdf]

DiD: Effect Heterogeneity

Victim Blaming Indicators

	Victim as Subject			Neg. of Offender Context		
female x sexual x post	-1.103			-0.236		
	(3.329)			(0.394)		
dem. judge x sexual x post		-2.099			-0.015	
		(3.729)			(0.225)	
dem. state x sexual x post			-0.276			-0.106
			(2.967)			(0.162)
post	X	X	X	X	X	X
court FE	X	X	X	X	X	X
# words	X	X	X	X	X	X

Table 1: DiD estimates of effect heterogeneity for victim blaming indicators. Significance levels: * p<0.1, ** p<0.05, *** p<0.01.
