# Supplementary material for: The impact of the #MeToo movement on language at court A text-based causal inference approach
Source: PLoS One. 2024 May 15;19(5):e0302827. doi: 10.1371/journal.pone.0302827 (PMC11095728; doi:10.1371/journal.pone.0302827)
Supplement: S6 Table — Text vectorization. (PDF) [file pone.0302827.s007.pdf]

DiD: Effect Heterogeneity

Text Vectorization

	BoW			Reduced Sample BoW			tf-idf		
female x sexual x post	0.891			1.170			-1.740		
	(1.171)			(1.194)			(2.207)		
dem. judge x sexual x post		0.340			0.764			-4.144	
		(1.938)			(2.145)			(2.993)	
dem. state x sexual x post			0.357			-0.152			2.169
			(1.335)			(1.207)			(1.841)
post	X	X	X	X	X	X	X	X	X
court FE	X	X	X	X	X	X	X	X	X
# words	X	X	X	X	X	X	X	X	X

Table 1: DiD estimates of effect heterogeneity for text vectorization-based opinion quantifiers. The distance of opinions to the H1 2015 average is expressed relative to the median distance of all opinions to the H1 2015 average, in percent. Significance levels: * p<0.1, ** p<0.05, *** p<0.01.
