# Supplementary figures and images for: The impact of the #MeToo movement on language at court A text-based causal inference approach
Source: PLoS One. 2024 May 15;19(5):e0302827. doi: 10.1371/journal.pone.0302827 (PMC11095728; doi:10.1371/journal.pone.0302827)

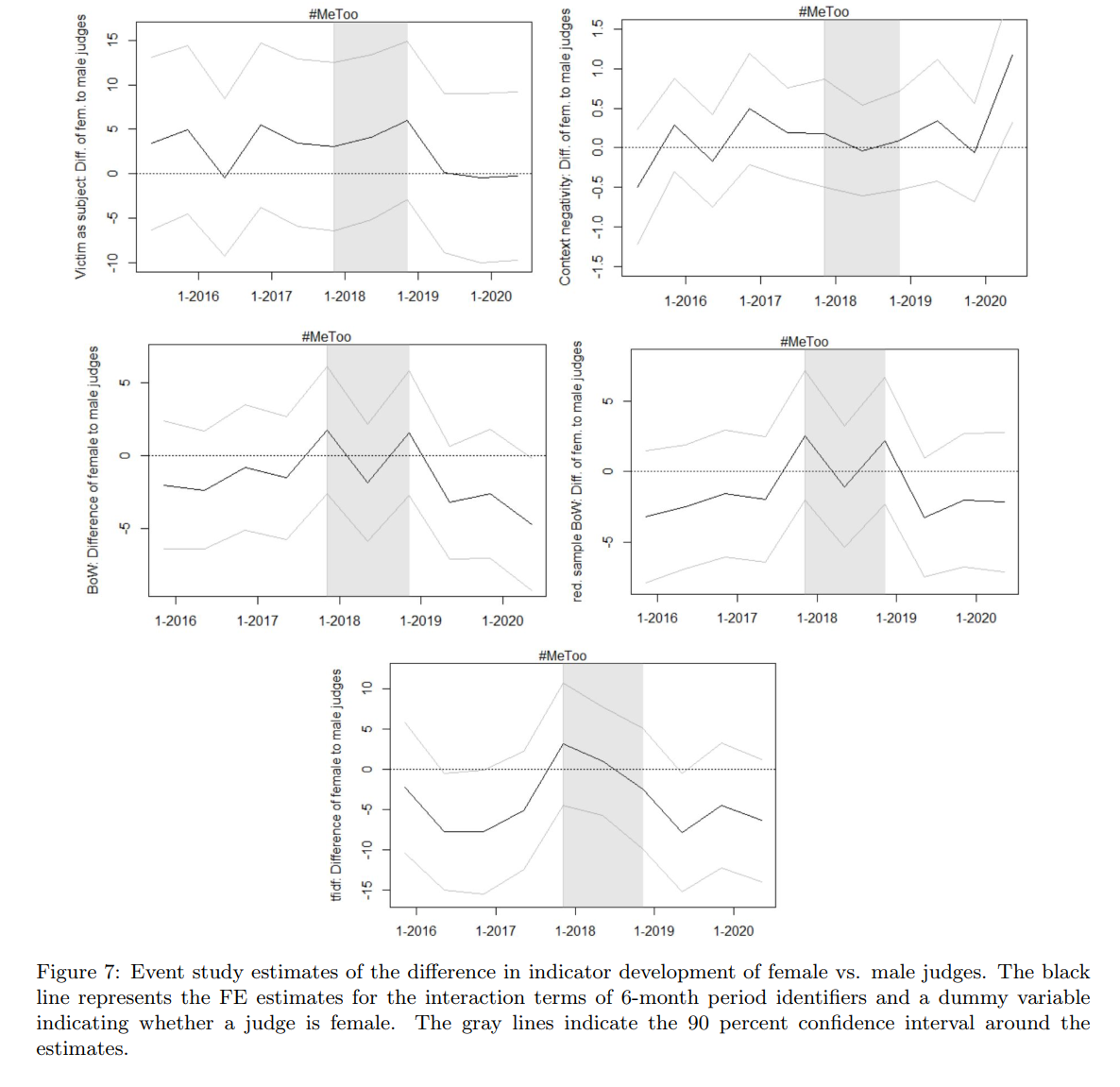

Supplement: S1 Fig — Event study estimates of difference in development of female vs. male judges. (TIF) [file pone.0302827.s008.tif]

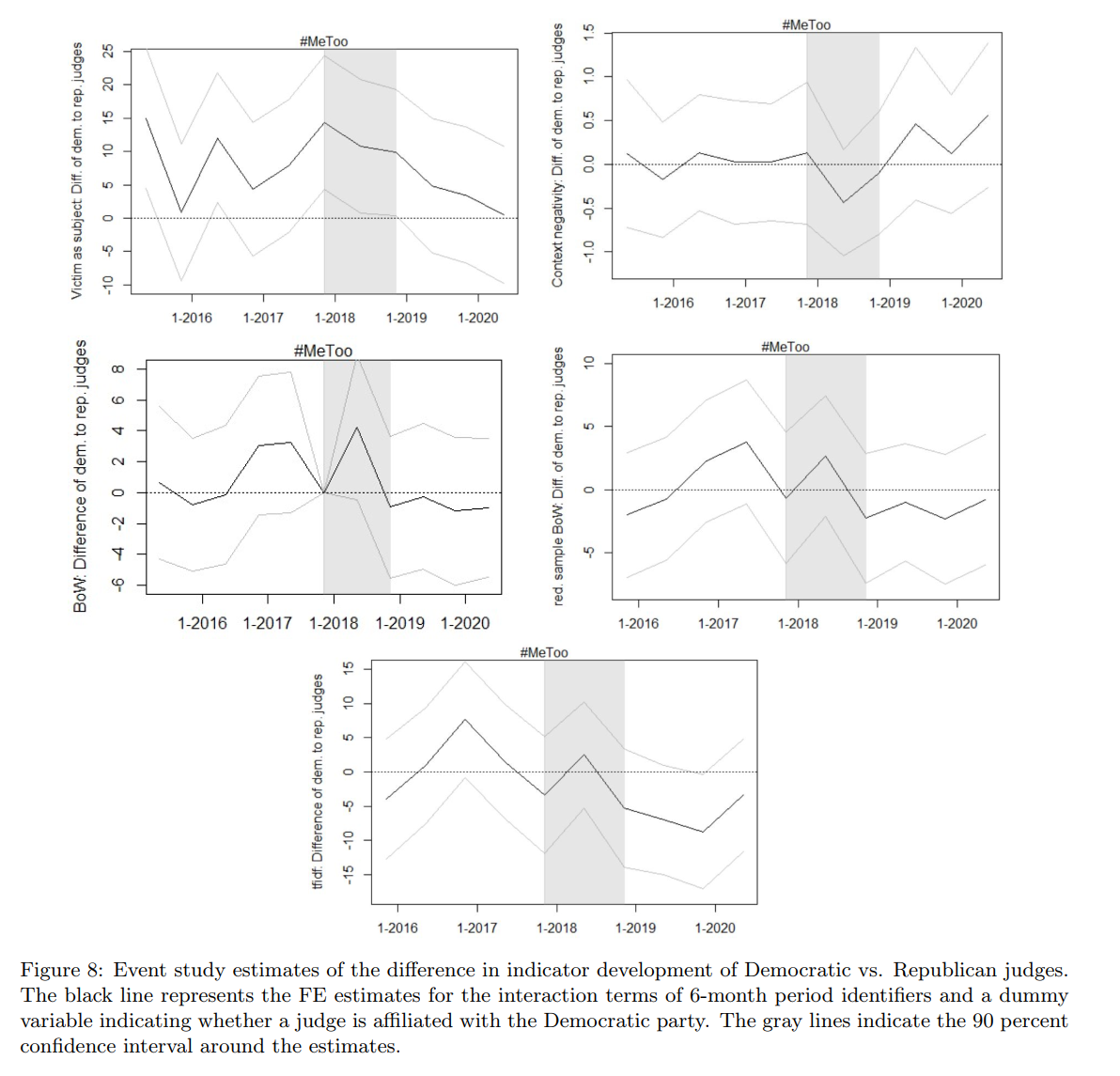

Supplement: S2 Fig — Event study estimates of difference in development of Democrats vs. Republicans. (TIF) [file pone.0302827.s009.tif]
